# Modification of Corneal Biomechanics and Intraocular Pressure Following Non-Penetrating Deep Sclerectomy

**DOI:** 10.3390/jcm11051216

**Published:** 2022-02-24

**Authors:** María Dolores Díaz-Barreda, Ignacio Sánchez-Marín, Ana Boned-Murillo, Itziar Pérez-Navarro, Juana Martínez, Elena Pardina-Claver, Diana Pérez, Francisco Javier Ascaso, Juan Ibáñez

**Affiliations:** 1 Department of Ophthalmology, Hospital Clínico Universitario Lozano Blesa, 50009 Zaragoza, Spain; nachosm89@gmail.com (I.S.-M.); anabomu@hotmail.com (A.B.-M.); ichuperez_87@hotmail.com (I.P.-N.); juanimarmor@hotmail.com (J.M.); elenapardina11@hotmail.com (E.P.-C.); dianapgpe@hotmail.com (D.P.); juanibanezalperte@msn.com (J.I.); 2 Department of Surgery, School of Medicine, University of Zaragoza, 50009 Zaragoza, Spain; 3 Aragon Health Research Institute (IIS Aragón), 50009 Zaragoza, Spain

**Keywords:** corneal biomechanics, ocular response analyzer, ORA, corneal hysteresis, glaucoma, tonometry, non-penetrating deep sclerectomy, Esnoper V-2000 implant

## Abstract

Changes in the cornea can influence outcomes in patients with primary open-angle glaucoma (POAG). We aimed to evaluate the relevance of changes in corneal biomechanics and intraocular pressure (IOP) in patients undergoing non-penetrating deep sclerectomy (NPDS) with the Esnoper V2000 implant^®^ (AJL Ophthalmic S.A., Gasteiz, Spain). We included 42 eyes of 42 patients with POAG scheduled for NPDS with the Esnoper V2000 implant. Biomechanical properties were measured by Ocular Response Analyzer^®^ G3 (ORA; Reichert Inc., Depew, NY, USA). Corneal hysteresis (CH), corneal resistance factor (CRF), corneal compensated IOP (IOPcc), and Goldmann-correlated IOP (IOPg) were measured the day before surgery and on day 1, 7, and 30 and 2 and 3 months after surgery. CH initially increased, fell below the presurgical value at 30 days after the surgery, and increased again at 2 and 3 months. CRF, IOPcc, and IOPg decreased on the first day after surgery, then followed a trend of increasing but stayed below pre-surgery levels. All values reached statistical significance. While observed changes in corneal biomechanics after NPDS and Esnoper V2000 implant were significant, more studies are needed if we are to understand their influence on corneal biomechanics and their clinical relevance in POAG.

## 1. Introduction

Glaucoma represents one of the main underlying causes of irreversible blindness worldwide, with the most frequent type being primary open-angle glaucoma (POAG) [1,2]. The main risk factor for disease progression, and the only one we can influence, is intraocular pressure (IOP); for this reason, its detailed study, along with the corneal properties (both structural and biomechanical) that can affect its measurement, is essential [3,4,5,6].

Goldmann applanation tonometry (GAT) is the gold standard in the measurement of the IOP, but the technique presents many inter-observer variations and is influenced by the curvature or central thickness of the cornea, biomechanical parameters of the cornea, and the age of the patient [7] (pp. 870–887) [8]. It has been shown, using a biomechanical model, that GAT does not always reflect true IOP values and that corneal compensated IOP (IOPcc) can become a fundamental parameter in the diagnosis and monitoring of this pathology [9]. Likewise, the resistance of the cornea to flattening by contact tonometry was the most determining factor to influence the differences in IOP between tonometers [6,10].

In an attempt to overcome the limitations of contact tonometers, other devices have been developed; the emergence of these new devices has resulted in new parameters, indices, and diagnostic algorithms that can help us to more quickly and reliably detect different pathological conditions [11]. One of the most recent is the Corvis^®^ ST (OCULUS Optikgeräte GmbH, Wetzlar, Germany). It is a classical non-contact tonometer combined with an ultra-fast Scheimpflug camera capable not only of giving more reliable IOP measurements but also of analyzing biomechanical properties of the cornea and its dynamic deformation [12].

In this paper we focus on the Ocular Response Analyzer^®^ G3 (ORA). Designed by Reichert Technologies (Reichert Inc., Depew, NY, USA), ORA is a non-contact device that measures, in vivo, the differential response of the cornea to applanation produced by a rapid air pulse over a period of approximately 20 milliseconds. By means of different parameters that we describe below, ORA provides information on the biomechanical and viscoelastic properties of the cornea [6,9]. Corneal hysteresis (CH) is a property that represents the dynamic resistance of the cornea to deformation (i.e., its ability to absorb and dissipate energy). The corneal resistance factor (CRF) represents static resistance and is proportional to the force applied to the cornea; CRF is related to the central corneal thickness (CCT), calculated by an ultrasonic pachymeter that forms part of the ORA, and the Goldmann-correlated IOP (IOPg). CRF is determined using the average of two IOP measurements made at the moment of maximum inward and outward applanation. A calculation is also made according to the air pressure required to flatten the central area of the cornea, using information provided by CH; this is known as the IOPcc. IOPcc offers several advantages over the IOP measured by GAT [7]. 

In recent years, the study of corneal biomechanics has been applied to the various branches of ophthalmology, including glaucoma [13]. Studies show that CH in subjects with glaucoma is significantly lower than in the general population [14,15,16]; this parameter has been associated with a greater defect in the optic disc. Further to this, a thinner layer of nerve fibers has been postulated as a risk factor for glaucoma progression, even in patients with well-controlled IOP measured by GAT [4,14,17,18,19,20]. In addition, the Ocular Hypertension Treatment Study (OHTS) concluded that CCT is a factor that can predict the evolution of ocular hypertension to POAG [21].

Studies also indicate that the continued use of certain prostaglandins (PGAs) can alter corneal biomechanics independent of the lowering of the IOP; this has also been observed in patients who had partial recovery of CH following therapy [22,23].

If we turn our attention to surgical treatments, there are a few isolated studies in the available literature that refer to biomechanical changes in the cornea following the different surgical techniques available to glaucoma patients. However, their limited number and heterogeneity make it very difficult to arrive at a conclusion; in fact, there is a general lack of studies on non-penetrating deep sclerectomy (NPDS), particularly in cases of implant-associated surgery, which is the technique of choice in our environment. 

The purpose of this study was to evaluate changes in the parameters of corneal biomechanics (CH and CRF) and IOP (PIOg and PIOcc) in patients with POAG undergoing NPDS surgery associated with Esnoper V-2000 implant^®^ (AJL Ophthalmic S.A., Gasteiz, Spain).

## 2. Materials and Methods

This was a consecutive non-randomized prospective study of 42 eyes corresponding to 42 patients diagnosed with POAG, selected for NPDS. It was carried out in the Department of Ophthalmology at the Lozano Blesa University Clinical Hospital, in Zaragoza, Spain, between September 2019 and July 2020. The study protocol was approved by the Review Committee of the Lozano Blesa University Clinical Hospital in Zaragoza and complied with Spanish legislation in the field of biomedical research; in the protection of personal data, Organic Law 3/2018 on the Protection of Personal Data; Basic Law 41/2002, regulating patient autonomy and rights; obligations regarding information and clinical documentation; and Law 14/2007 on biomedical research. All the research was carried out following the principles of the Declaration of Helsinki, and all patients signed an informed consent (IC) and were given a copy of it. 

The inclusion criteria were as follows: The patient must be over 18 years old and present a diagnosis of POAG (requirement was for reproducible defects in the visual field (VF) detected by automated perimetry with Humphrey^®^ 3 Field Analyzer (Carl Zeiss Meditec, Inc., Dublin, CA, USA) strategy 24-2 and corresponding defect in the retinal nerve fiber layer (RNFL) in the swept-source-optical coherence tomography (DRI OCT Triton™, Topcon, Tokyo, Japan); POAG had to have been treated for a minimum of the previous six months with at least two topical anti-glaucomatous drugs, one of which was supposed to be a PGA eye drop; despite topical treatment, the patient should not achieve adequate IOP control and the progression of POAG should continue, with the patient therefore a candidate for NPDS without associating phacoemulsification; no previous history of pathologies that could affect the cornea and no signs of retinopathy or optic neuropathy other than glaucoma; images obtained had to have a quality score higher than 20.

The exclusion criteria were as follows: The patient could not have a personal history of any ophthalmological condition other than glaucomatous damage caused by POAG (except senile cataract without surgery criteria); extreme axial lengths (below 22 mm and above 26 mm); surgery on any eye or have had an ocular trauma that required consultation with an ophthalmologist; any type of intraoperative complication, such as perforation of Descemet’s membrane and conversion to trabeculectomy, or any postoperative complication; presentation of an IOP lower than 5 mmHg by GAT after surgery was also an exclusion criterion. Patients whose tests were not of sufficient quality to be analyzed were also excluded, as were patients who had required non-topical IOP lowering medication in the 6 months prior to surgery and those requiring topical medication (including hypotensive drops) three months after surgery (not counting the drops included in the post-surgical protocol).

Although 53 eyes of 53 patients with POAG were initially included, 1 was ruled out because of myopia magna with an axial length of 27.02 mm (not detected in the initial interview), 8 were ruled out for not meeting the time requirement for treatment with topical eye drops (also not recorded in the first interview), and 2 were ruled out for needing hypotensive eye drops after surgery. Finally, the data of 42 eyes of 42 patients were analyzed. 

All surgeries were performed by the same surgeon (J.I.), as explained below. A fornix-based conjunctival flap was performed, followed by a superficial scleral flap of approximately one-third of the total thickness. A portion of 0.02% mitomycin C (MMC), prepared in the hospital pharmacy, was used for 2 min at both the scleral and subconjunctival levels. Finally, a small, deep flap was created, leaving a thin sheet of sclera to plane of the Schlemm’s canal which was dissected. Esnoper V-2000 (AJL^®^) was used as a suprachoroidal implant without sutures. Finally, the surface flap was sutured with 10/0 Nylon (Dafilon^®^, B. Braun Surgical S.A., Barcelona, Spain) and the conjunctival flap with 8/0 Silk (Silkam^®^, B. Braun Surgical S.A., Barcelona, Spain). Postoperatively, patients followed a downward regimen of TobraDex^®^ eye drops (Novartis, Basel, Switzerland) (1 mg of dexamethasone and 3 mg of tobramycin) according to postsurgical protocol.

The biomechanical properties of the cornea were measured with ORA: three measurements were made, and the mean all of them was expressed in millimeters of mercury (mmHg), calculated for the analysis. Values measured were CH, CRF, IOPcc, and IOPg; all were taken by the same ophthalmologist in the morning and in a time range of 3 h. All measurements were made the day before the surgery and on day 1, 7, and 30 and at 2 and 3 months after surgery.

### Statistical Analysis

The statistical analysis was carried out using IBM SPSS v.23 (IBM, Armonk, NY, USA), calculating the means and standard deviations (SD), medians, and ranges for each variable and each time moment. To check the normality of the data to assess whether to apply the parametric or non-parametric tests corresponding to each analysis, the Kolmogorov–Smirnov test was used for a sample, obtaining significant deviations from the normal curve in most variables.

A value of *p* < 0.05 was used to consider the result statistically significant. The statistics were calculated using MedCalc ver.15.2 (MedCalc Software Bvba, Ostend, Belgium).

A global analysis was carried out for each variable using the Friedman test for non-parametric dependent or related samples to evaluate the differences in biomechanical parameters over time (day before surgery, and at day 1, 7, and 30 and at 2 and 3 months after surgery).

## 3. Results

The study sample consisted of 42 eyes from 42 different patients. The median age of the sample was 68.19 +/− 11.88 years. The demographic characteristics are set out in Table 1.

**Figure 1:**
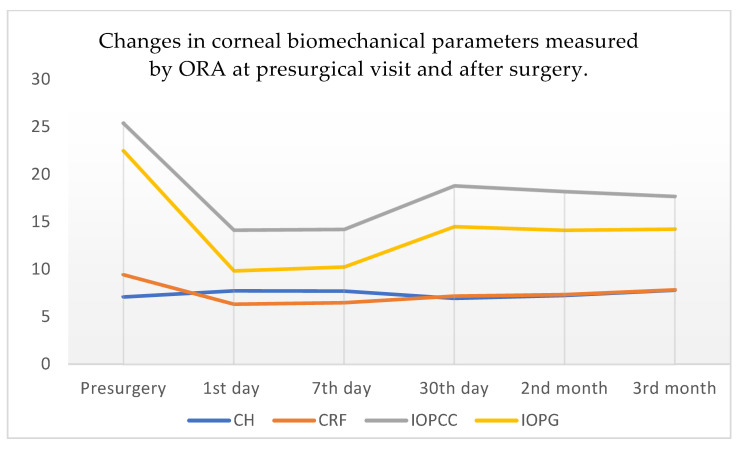
Data on biomechanical characteristics obtained by ORA before surgery and at days 1, 7, and 30 after surgery and at 2 and 3 months after surgery. The vertical axis on the left corresponds to mmHg, while the horizontal axis refers to time. The color legend for each variable studied is placed below. CH: corneal hysteresis; CRF: corneal resistance factor; IOPcc: compensated intraocular pressure; IOPg: Goldmann-correlated intraocular pressure.

Table 2 shows the data on the changes in the ORA variables (CH, CRF, IOPcc, and IOPg) at the different study visits. These data are supported by Figure 1, where the four variables are shown across time. Regarding the presurgical values, CH increased on days 1 and 7, descending below the presurgical value at day 30, after which it increased again at 2 and 3 months after the intervention to above the values prior to surgery. The rest of the variables (CRF, IOPcc, IOPg) decreased the first day after surgery, then followed an increasing trend but stayed below pre-surgical levels. All values reached statistical significance. 

## 4. Discussion

IOP is the only risk factor in the development and progression of glaucomatous optic neuropathy that can be treated at the present time. It is essential to obtain a reliable measurement that allows us to reach a correct diagnosis and classification for the proper management and follow-up of the glaucoma patient. Nowadays, GAT is the gold standard. However, it is based on Imbert–Fick’s law, which assumes conditions that are not real, such as that the cornea has a radius of constant curvature, which is always spherical, with minimal thickness, and that presents the same rigidity in all cases [24,25]. Further to this, a low degree of reproducibility of this measure has been demonstrated due to its interobserver variability [26]. In order to overcome these drawbacks, other devices have been developed, among which the ORA stands out. ORA is a non-contact instrument that provides a reproducible measurement of IOP that is not influenced by the person performing the test and that, perhaps most importantly, is based on biomechanical properties and parameters of the cornea [6,25]. Therefore, the ORA not only helps us to study and understand the properties of the cornea but also allows us to quantify the properties numerically. This allows us to compare the results obtained in order to standardize and look for ranges of normality, with which we can detect patients who deviate from them, and whose disease may be progressing due to the limitations of other devices. 

Because ORA is a relatively new technology, available literature is limited. It is important to emphasize that in many studies, including ours, it is impossible to determine what proportion of the observed changes in corneal biomechanics and IOP are exclusively due to each of the interventions performed. New research would need to be designed in such a way as to isolate each factor that may affect these parameters. 

In an attempt to address this issue, Touboul et al. [15] published a prospective study in 2008 in which they looked for correlations between the data provided by ORA across four different ophthalmological pathologies that they grouped into four groups: glaucoma (*n* = 159), keratoconus (*n* = 88), laser-assisted laser in situ keratomileusis (LASIK) (*n* = 78), and photorefractive keratectomy (PRK) (*n* = 39) (all vs. a control group without ophthalmic pathologies (*n* = 122)). The authors found statistically significant differences between GAT and IOPcc and IOPg in all pathological groups. It was also found that, in the general population, the higher the CH, the closer the values of GAT and IOPcc. CRF maintained similar values in the glaucoma group vs. the control group, while CH was lower and seemed independent of age. 

Focusing on glaucomatous pathology, we know is that eyes with POAG show certain corneal characteristics that could also affect other structures of the eyeball. This could translate into a special susceptibility to increases in IOP at the level of structures such as lamina cribosa [27]. A significant decrease in CH is observed in patients with glaucoma (especially in cases of congenital glaucoma). With regard to CRF, elevated values were found in all glaucoma suspicion groups in different studies [25,28]. Of special interest is the research of Del Buey et al. [14], which analyzed 1065 eyes; the group describe a lower CH with a statistically significant difference in patients with glaucoma compared with healthy eyes. Additionally statistically significant was the difference with respect to IOPg and IOPcc in the group of patients with glaucoma and controls. Unexpectedly, the CRF was superior in all pathological groups with respect to control, but it was only significant in groups suspected of glaucoma, not in glaucoma patients. The data collected in our study were consistent with those discussed above, presenting a CH even lower with the IOPcc and the IOPg slightly higher. Perhaps the degree of severity of glaucoma can influence the parameters measured by ORA, our representative sample being of patients with moderate–severe glaucomatous damage with indication of surgery after the failure of other treatments and, therefore, being able to present more extreme averages than the group of glaucoma presented by Del Buey et al. [14].

Regarding eye surgery, the study of corneal biomechanical properties has centered mostly on phacoemulsification, and results have been obtained in different studies that indicate a decrease in CH values and an increase in CRF [24,29,30]. However, the evidence after glaucoma surgery is not very broad, despite being fundamental to understanding the intrinsic changes that may occur, and offers little evidence in terms of what real benefit surgical intervention will bring to the patient. 

Our findings reflect those described for other glaucoma ophthalmological surgeries, such as those presented by Pakravan et al. [31]. Pakravan et al. found a significant increase in both CRF and CH in all groups of glaucomatous patients who were studied at 3 months following different surgeries (trabeculectomy with MMC (*n* = 23 eyes); trabeculectomy with MMC with phacoemulsification (*n* = 17 eyes)); Ahmed valve implantation (*n* = 17); cataract only in non-glaucomatous patients (*n* = 26 eyes)). This calls into question a possible relationship between the decrease in IOP (measured by GAT) and the increase in CH after treatment, which could be the first hypothesis to be considered for understanding the results of our study. The only ones who have described a weak correlation between both parameters, and only preoperatively, are Iordanidou et al. [32] and Sun et al. [22], who argue that ORA could make a mistake with the measurement of high pressures. It has even been shown that the change that occurs over 24 h in IOP does not affect biomechanical properties [33]. Sun et al. [22] analyzed a group of 40 patients with unilateral POAG who underwent trabeculectomy, achieving a statistically significant increase in CH only 2 weeks after the intervention. It would be comparable with what we obtained on day 1 and 7 after surgery. Unlike ours, one month after surgery its results do not reach statistical significance, although they remain above preoperative values, at which time we observed a marked decrease in CH. We should consider whether the changes produced by trabeculectomy (penetrating filter surgery) on corneal biomechanics are really more stable one month after the intervention than those caused by NPDS, therefore maintaining the upward trend despite not reaching statistically significant values. 

If we search the literature for studies describing changes in corneal biomechanics through ORA produced by implant-associated surgery, there are very few results. Konstantinidis et al. [34] published a prospective study to compare corneal biomechanical changes in two groups: group 1 of patients with glaucoma who had an EX-PRESS^®^ (Novartis, Basel, Switzerland) device implanted (*n* = 19) and group 2 who underwent a trabeculectomy (*n* = 11). Measurements were made of the eyes operated with ORA preoperatively and after surgery in months 1, 6, and 12. CH increased significantly for months 6 and 12 in group 1 and for all postoperative measures in group 2, compared with those obtained pre-surgery. Regarding the CRF parameter, it decreased significantly for both groups in all measures. Konstantinidis et al. found no correlation between CH and CRF. These results support our hypothesis that CH and CRF will follow the respective increasing and decreasing trend that we have found in our study. Similar changes between the two groups lead us to think that the introduction of an implant could have an influence similar to a piercing technique, although the sample size of each group was too small to validate this hypothesis by itself. On the other hand, it could be supported by the data provided by Casado et al. [35], which did not show statistically significant differences between two groups: the first formed by 20 eyes of 20 patients who underwent NPDS with implantation (Aquaflow; Staar Surgical AG, Nidau, Switzerland) and the second group by 20 eyes of the same 20 patients (the contralateral), with an intervention of sclerectomy converted to trabeculectomy. Group 1 had lower values (still reaching statistical significance) for both CH and CRF, which could be associated with a better prognosis due to worse results, which have been demonstrated in the visual field associated with lower CH values [17,20].

Special attention should be given to the work published by Iordanidou et al. [32], who were the first to use ORA technology to analyze 30 eyes of 30 patients with POAG to evaluate the biomechanical changes produced after an NPDS with a collagen implant (Staar Surgical AG, Nidau, Switzerland). They carried out a measurement on day 1 and 8, and at 1 month after surgery. Of the parameters studied, the only one whose variation remained statistically significant was CH, which increased the day after the surgery from 7.51 +/− 1.56 mmHg (in our study it was 7.10 +/− 2.02 mmHg) to 9.38 +/− 1.77 mmHg (7.74 +/− 1.96 mmHg), where it remained on day 8 with a value of 9.2 +/− 1.57 mmHg (in our case, on day 7 it was 7.72 +/− 1.75 mmHg) and then decreased to the figure of 8.41 +/− 1.72 mmHg per month after the NPDS (6.95 +/− 1.84 mmHg). The curve that CH draws was repeated in our study, presenting minor changes, although it also reached statistical significance, reaching the month of surgery, to descend below the preoperative level. Unlike Iordanidou et al. [32], who only follow up until the 30th postoperative day, we continued follow up for three months, analyzing the cases on days 1, 7, and 30 and at 2 and 3 months. We observed an increase in CH values at 2 and 3 months. In this study, IOPg had a preoperative mean value of 19.57 +/− 6.32 mmHg, which was drastically reduced the next day to a value of 5.2 +/− 3.49 mmHg, reaching statistical significance. Subsequently, IOPg began to increase without reaching statistical significance, reaching 8.32 +/− 5.37 mmHg on day 8 of the intervention and 12.71 +/− 7.43 mmHg one month after surgery. IOPcc and CRF initially decrease, to gradually increase without reaching the preoperative value, which makes us think that with a larger sample size or a longer monitoring time, these results could have reached statistical significance and support ours. 

Following this same line, Díez-Álvarez et al. [19] published a prospective study of 49 patients with a mean age of 73.5 +/− 8.2 years who had been on anti-glaucomatous eye drops (77.6% with PGAs) and who were intervened with NPDS in combination with phacoemulsification (NPDS + P) in 26 cases or with and NPDS alone in the other 23 cases. The study analyses corneal biomechanics with ORA 3 months after surgery. Unlike the study presented above and ours, in no case did they accompany the surgery of an implant, which makes it difficult for us to compare results. Despite this, it presents a sample of size, age, and biomechanical data prior to surgery similar to ours. They performed a single postoperative measure, observing in both groups an increase in CH and a decrease in CRF, IOPcc, and IOPg. All measurements reached statistical significance and were consistent with our results. Based on their results, and despite preoperative values, the postoperative reduction in IOP was the independent factor that most influenced optic nerve changes after surgery. 

We would like to point out that the first-month CH in our results followed a curve equal to that defined by Iordanidou et al. [32]; first ascends, and then descends, but after 3 months we find values greater than presurgical ones, as Díez-Álvarez et al. [19] also describe. This change in trend could be justified, or could at least be altered, by taking into account that an increase in CH associated with the use of PGAs has been demonstrated without being related to a decrease in IOP by GAT, by influencing extracellular matrix remodeling and modification of corneal properties [23,36,37]. In our study, all the participants had taken PGAs as a treatment prior to surgery, and PGAs are able to maintain their effect on corneal biomechanics after suspension for a few days and then gradually disappear [37]. On the other hand, the changes during the month after surgery could be altered by the use, according to the protocol, of eye drops with dexamethasone (TobraDex^®^) since it has been shown that it can increase IOP, although we have not found data in the literature on its effects on corneal biomechanics [38]. 

Our study has certain limitations, starting with the sample size, which might be limited due to the strict inclusion criteria of the study, which were put in place to ensure as homogeneous and reproducible a sample as possible, while avoiding as many confounding factors as possible. Another major limitation to highlight is the difficulty of comparing our results with other published studies. There are important works that establish biomechanical values in the healthy population, but not in a standardized way by age and sex groups. While we are able to find research in the literature on corneal biomechanics and glaucoma, the studies were quite heterogeneous in their methods and results. Regarding surgeries, the diversity was even greater since there are many techniques and devices to which the intrinsic variability is added with each surgeon who performs the procedure. In this sense, performing two different surgeries on the eyes of the same patient would be ideal to be able to compare them and thus establish differences, as Casado et al. [35] have reported. In the case of filtering surgery, it is necessary to consider the criteria of choice of each technique, which is usually determined by glaucomatous damage and its ability to decrease tension. Sclera deserves special consideration since it provides the anatomical support of the eye for many measurements we make, such as in the measurement of IOP by GAT and measurements by ORA. Therefore, surgeries such as trabeculectomy, in which the sclerectomy that is performed produces a thinning of the cornea–sclera interface, could clearly affect corneal biomechanics and its measurements. It has already been seen that structural changes of the eyeball affect its biomechanical properties, as described by Grost-Otero et al. [39] in a study in which they analyzed 20 patients who underwent surgery for pterygium in one eye and compared with the contralateral eye, finding a statistically significant decrease in CH in the first group with respect to the second. In addition, we must consider that there may be anatomical changes that are not completely restored, or that restore very slowly, after glaucoma surgery. That could be in favor of obtaining similar results, regardless of the technique or device used. Another limitation would be the failure to collect and analyze the data on CCT, axial length, and refraction, as these would be of interest. Regarding topical anti-glaucomatous hypotensive eye drops, there is evidence that affirms how PGAs influence corneal biomechanics, but evidence does not differ between the type of PGAs used, and the duration of treatment, or IOP prior to PGAs use [22,36]. The use of MMC (including duration and dose), as well as the use of an implant and in which location it is located, should also be considered. As we have already highlighted at the beginning of the discussion, other limitations would be that we were unable to determine what percentage of the biomechanical changes were due exclusively to the surgery, the implant, the use of MMC during surgery, the use of topical PGAs prior to surgery and its subsequent discontinuation, and the use of TobraDex^®^ afterwards. 

We believe our findings represent the first published study of which we are aware on variations in corneal biomechanics after an NPDS intervention, the surgery of choice in our environment, with Esnoper V-2000 implant, and the first whose evolution has been collected and analyzed throughout the first three post-surgical months. This would imply a greater sample size and a longer follow-up period than comparable studies presented in the existing literature, establishing how corneal biomechanics varies between the values before and after surgery.

## 5. Conclusions

According to our analysis, we conclude that at 3 months of follow-up, CRF remain below preoperative values, and CH above, after having decreased in the first month, reaching statistical significance in all measures. After the NPDS, as expected, the IOP was successfully maintained below preoperative values, being assessed by the IOPcc and IOPg values provided by the ORA.

More research is needed following this line with a larger sample size and longer follow-up periods and with more homogeneous groups in terms of age, glaucomatous damage, pre-surgery treatment, and surgical technique to evaluate the changes caused in corneal biomechanics and their relevance in clinical practice.

## Figures and Tables

**Table 1:** Demographic characteristics of the 42 patients analyzed.

CHARACTERISTICS		VALUES
**SEX, *N* (%)**	Women	12 (28.57)
	Men	30 (71.43)
**AGE, YEARS**	Mean +/−SD	68.19 +/− 11.88 years
	Median (range)	70 (45–70)
**LATERALITY OF THE TESTED EYE, *N* (%)**	Right	21 (50)
	Left	21 (50)
**NUMBER OF TOPICAL ANTIHYPERTENSIVE EYE DROPS PRIOR TO SURGERY, *N* (%)**	1	0 (0)
	2	4 (9.53)
	3	21 (50)
	4	17 (40.47)

SD: standard deviation.

**Table 2:** Data on biomechanical characteristics obtained by ORA day before surgery and at day 1, 7, and 30 and at 2 and 3 months after surgery. All data are measured in mmHg, with the mean and standard deviation (SD) in brackets on the first line. The second line shows the median and the range in brackets.

	*N*	*Presurgery*	*1st Day*	*7th Day*	*30th Day*	*2nd Month*	*3rd Month*	*p*
***CH***	42	7.10 (2.02) 7.3 (3.1–12.5)	7.74 (1.96) 7.9 (1.3–13.9)	7.72 (1.75) 7.35 (4.4–11)	6.95 (1.84) 6.9 (2.1–10.7)	7.25 (1.46) 7.55 (4.4–10.3)	7.81 (1.55) 8.15 (4.8–11.8)	0.031
***CRF***	42	9.44 (1.97) 9.25 (5.6–14.49)	6.34 (1.94) 6.45 (2.7–9.4)	6.5 (2.12) 6.5 (2.7–12.2)	7.19 (2.06) 6.65 (4.1–14)	7.35 (1.94) 7.35 (3.8–13.9)	7.85 (1.93) 7.2 (4.7–15.1)	<0.001
***IOPcc***	42	25.38 (7.48) 23.5 (11.2–43)	14.14 (9.03) 12.25 (0.2–42.7)	14.21 (6.75) 12.85 (4.7–32.2)	18.81 (7.69) 17.75 (6.2–43.5)	18.2 (5.82) 16.65 (6.2–36.9)	17.68 (6.09) 16.75 (6.3–31.9)	<0.001
***IOPg***	42	22.49 (7.47) 20.55 (11.9–39.1)	9.85 (7.63) 8.5 (5.1–30.7)	10.25 (6.78) 8.85 (5.0–26.8)	14.51 (7.54) 12.8 (5.4–31.3)	14.14 (6.1) 12.35 (5.2–25.9)	14.24 (6.24) 12.55 (5.9–26.1)	<0.001

CH: corneal hysteresis; CRF: corneal resistance factor; IOPcc: compensated intraocular pressure; IOPg: Goldmann-correlated intraocular pressure.
